# A small molecule PKC**ε** inhibitor reduces hyperalgesia induced by paclitaxel or opioid withdrawal

**DOI:** 10.1172/jci.insight.186805

**Published:** 2025-04-22

**Authors:** Adriana Gregory-Flores, Ivan J.M. Bonet, Stève Desaivre, Jon D. Levine, Stanton F. McHardy, Harmannus C. de Kraker, Nicholas A. Clanton, Peter M. LoCoco, Nicholas M. Russell, Caleb Fleischer, Robert O. Messing, Michela Marinelli

**Affiliations:** 1 Institute for Neuroscience and; 2 Department of Neuroscience and the Waggoner Center for Alcohol and Addiction Research, University of Texas at Austin, Austin, Texas, USA.; 3 Department of Oral and Maxillofacial Surgery, UCSF Pain and Addiction Research Center, and; 4 Department of Medicine, Division of Neuroscience, UCSF, San Francisco, California, USA.; 5 Department of Chemistry, Center for Innovative Drug Discovery, and; 6 Voelcker Preclinical Pharmacology Core, Department of Chemistry, University of Texas at San Antonio, San Antonio, Texas, USA.; 7 Division of Pharmacology and Toxicology, College of Pharmacy,; 8 Department of Neurology and the Mulva Clinic for the Neurosciences, Dell Medical School, and; 9 Department of Psychiatry and Behavioral Sciences, Dell Medical School, University of Texas at Austin, Austin, Texas, USA.

**Keywords:** Neuroscience, Therapeutics, Addiction, Pain, Protein kinases

## Abstract

The enzyme protein kinase C ε (PKCε) plays an important role in pain signaling and represents a promising therapeutic target for the treatment of chronic pain. We designed and generated a small molecule inhibitor of PKCε, CP612, and examined its effect in a rodent model of chemotherapy-induced neuropathic pain produced by paclitaxel, which does not respond well to current therapeutics. In addition, many patients with chronic pain use opiates, which over time can become ineffective, and attempts to discontinue them can increase pain thereby promoting sustained opioid use. Therefore, we also investigated if CP612 alters pain due to opioid withdrawal. We found that CP612 attenuated hyperalgesia produced by paclitaxel, and it both prevented and reversed hyperalgesia induced by opioid withdrawal. It was not self-administered and did not affect morphine self-administration. These findings suggest that inhibition of PKCε is an effective, nonaddictive strategy to treat chemotherapy-induced neuropathic pain, with the added benefit of preventing increases in pain that occur as opioid treatment is discontinued. This latter property could benefit individuals with chronic pain who find it difficult to discontinue opioids.

## Abstract 2

We designed and developed an inhibitor of the enzyme PKCε that reduces pain induced by cancer chemotherapy and prevents and reverses pain during opioid withdrawal.

## Introduction

According to the Centers for Disease Control and Prevention, an estimated 50 million adults in the United States experience chronic pain (1), with an estimated annual cost exceeding $600 billion (2, 3). The quality of life for these individuals is impaired due to suffering, disability, and unemployment.

A class of pain syndromes that is particularly difficult to treat is that resulting as a common adverse effect of cancer treatment: chemotherapy-induced peripheral neuropathy (CIPN) (4, 5). CIPN adversely affects the quality of life in oncology patients due to discomfort, pain, disability, and risk of falling. With cytotoxic agents such as the chemotherapeutic agent paclitaxel, pain is common and can be persistent with approximately half of patients having symptoms 1 year after treatment (4, 6). Unfortunately, pain associated with CIPN is poorly responsive to available medications, and the only agent recommended by the American Society of Clinical Oncology is duloxetine, which has only a modest effect (4, 5). Moreover many patients with CIPN or other causes of chronic pain receive long-term opioid therapy, which can become ineffective over time and difficult to taper since reducing the dose of opioid medications can worsen pain (7–9). Because of these limitations in the treatment of chronic pain, there is a major need for developing new, effective, nonopioid pain medications.

Protein kinase C is a family of 10 serine-threonine kinases that transduce signals carried by lipid second messengers (10). A large body of literature indicates that 1 PKC isoform, PKCε, is particularly important for signaling in peripheral nociceptive sensory neurons and that this kinase mediates inflammatory pain, neuropathic pain, and the transition from acute to chronic pain (11–13). Such findings have sparked interest in developing small molecule inhibitors of PKCε to treat chronic pain.

We previously reported the design of PKCε inhibitors based on Compound 397 (described in patent WO 2007/006546), which was developed from the ROCK inhibitor Y-27632. The lead compound we initially developed (Compound 1) inhibited PKCε but enhanced the hypnotic effect of ethanol in *Prkce*-null mice, indicating an off-target effect (14). Compound 1 also transiently depressed locomotion in association with reversible hypotension (14). We suspected these effects were due to ROCK inhibition since ROCK inhibitors can cause hypotension (15, 16) and Compound 1 inhibited ROCK1 and PKCε with similar potency.

To improve selectivity and potency for PKCε, we designed and generated a small molecule with drug-like properties that potently inhibits PKCε and investigated if it reduces hyperalgesia due to paclitaxel CIPN or opioid withdrawal in rats. To determine if it carries addictive potential, we also investigated if it would be self-administered or would increase opioid self-administration.

## Results

### Characterization of the PKCε inhibitor CP612.

**Figure 1:**
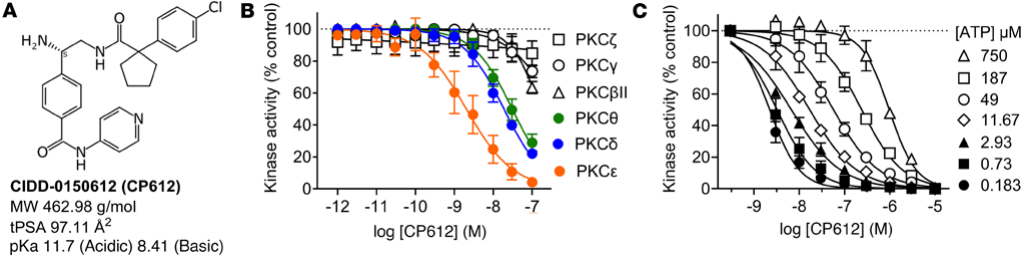
Structure and characterization of CP612. (**A**) Structure and characteristics of CP612. tPSA, topological polar surface area. (**B**) CP612 inhibited novel PKCs by > 50% at 30 nM but was more potent against PKCε (IC_50_ = 2.03 nM) than PKCδ (IC_50_ = 18.8 nM) or PKCθ (30.7 nM). (**C**) Dose-response curves for inhibition of PKCε by CP612 showing a rightward shift at higher [ATP]. Data are shown as mean ± SEM (*n* = 3 experiments each done in duplicate or triplicate).

We developed an efficient enantioselective synthesis and carried out structure-activity-relationship studies that led to the development of CIDD-0150612 (CP612; Figure 1A), to improve selectivity and potency for PKCε. CP612 possessed physicochemical properties known to be favorable for CNS drugs (17). These include kinetic aqueous solubility of 7.41 ± 0.12 μM and logD of 1.50 ± 0.75. CP612 showed high plasma protein binding (89.78% ± 1.65%) but good plasma stability and a low microsomal clearance, suggesting low first-pass hepatic metabolism (Supplemental Table 1; supplemental material available online with this article; https://doi.org/10.1172/jci.insight.186805DS1).

CP612 inhibited PKCε with IC_50_ = 1.9 ± 0.4 nM (*n* = 42) and ROCK1 with IC_50_ = 65.9 ± 10.8 nM (*n* = 42) making it approximately 55-fold more selective against PKCε over ROCK1. CP612 was screened for kinome specificity at 200 nM against 468 kinases using the scanMax assay panel from Eurofins-DiscoverX. CP612 inhibited 10 other WT kinases besides PKCε, all to < 10% of control: CLK1 (CDC Like Kinase 1), CLK4, PKN1 (Protein kinase N1), PKN2, PKCδ, PKCη, PKCθ, PRKG2 (Protein Kinase CGMP-Dependent 2), ROCK1, and ROCK2. When assayed against several PKC isozymes, CP612 did not inhibit atypical PKCζ, weakly inhibited conventional PKCβII and PKCγ and, within the nPKC subfamily, was most potent against PKCε (Figure 1B).

Since CP612 was derived from Compound 1 and Y-27632, we predicted it would be a competitive inhibitor of ATP binding to PKCε. Therefore, we assayed PKCε activity with increasing concentrations of ATP and in the presence of increasing concentrations of CP612. The EC_50_ for ATP was 4.2 μM (2.23–7.61 μM, 95% CI) and it increased 40-fold to 168 μM (121–250 μM, 95% CI) in the presence of 300 nM CP612 (Figure 1C). This rightward shift indicates that CP612 inhibits PKCε by competing with ATP.

**Figure 2:**
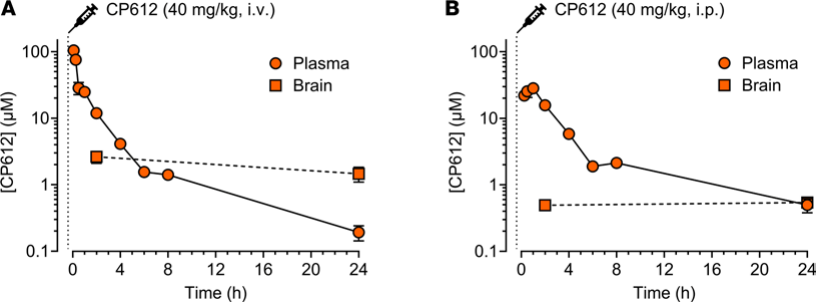
Pharmacokinetics of CP612. (**A** and **B**) Concentrations of CP612 (40 mg/kg) in male mice after i.v. or i.p. administration declined over time in plasma but remained more stable in the brain up to 24 hours afterward. Data are shown as mean ± SEM (*n* = 2–6 per group).

We next performed a pharmacokinetics study in rats, which showed that CP612 (40 mg/kg) has a plasma half-life of 4.0 ± 0.6 hours after i.v. administration and 6.8 ± 0.7 hours after i.p. administration (Figure 2). The C_max_ was 104.5 ± 3.1 μM at the earliest time point (5 minutes) after i.v. administration and was 29.3 ± 1.0 μM between 30 and 60 minutes after i.p. administration. Because binding to plasma protein was approximately 90%, we predicted that the free concentration at each time point was 10 times lower than the measured plasma concentration. After i.v. administration, levels of CP612 decreased between 2 hours and 24 hours by about 70-fold in plasma but only by less than 2-fold in the brain (Figure 2A), resulting in an increase in the brain/plasma concentration ratio from 0.16 ± 0.03 at 2 hours to 9.17 ± 3.19 at 24 hours. After i.p. administration, levels of CP612 decreased between 2 hours and 24 hours by about 30-fold in plasma but remained stable in the brain (Figure 2B), resulting in an increase in the brain/plasma concentration ratio from 0.03 ± 0.009 at 2 hours to 1.18 ± 0.42 at 24 hours. These results indicate that CP612 enters the brain and is cleared from the brain at a much slower rate than from plasma.

### CP612 reduces PKCε-dependent hyperalgesia.

**Figure 3:**
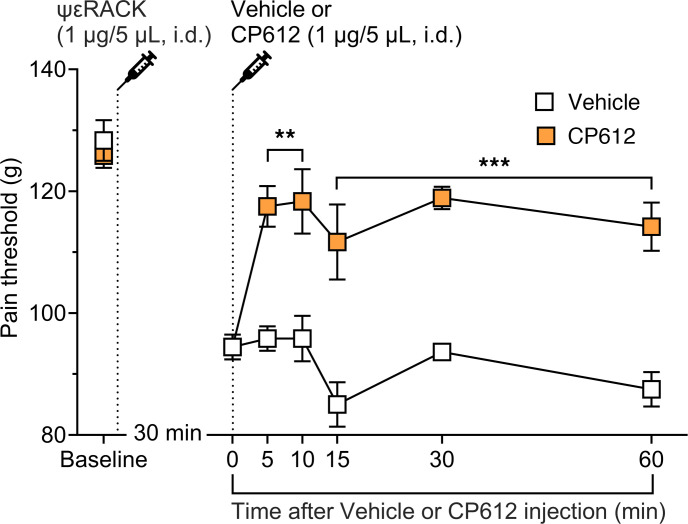
Hyperalgesia induced by the PKCε activator ψεRACK. Administration of the PKCε activator ψεRACK (1 μg/5 μL, i.d.) induced hyperalgesia in male rats. This was attenuated by CP612 (1 μg/5 μL, i.d.), up to 60 minutes after its administration. Data are shown as mean ± SEM (*n* = 6 paws per group). **P < 0.01, ***P < 0.001 compared with the same time points in the Vehicle group using Tukey’s post hoc test.

To directly test if CP612 inhibits PKCε-dependent hyperalgesia, we administered the highly selective PKCε activator, ψεRACK, into the dorsum of the hind paw (1 μg/5 μL, intradermally) of rats. Thirty minutes later, rats were administered vehicle (5 μL, intradermally) or CP612 (1 μg/5 μL, intradermally) (Figure 3). We measured nociceptive thresholds at the same site, at baseline, 30 minutes after the administration of ψεRACK (time 0) and 15, 30, and 60 minutes after the administration of vehicle or CP612. Pain thresholds at baseline and at time 0 did not differ across groups (*P* = 0.999 and *P* = 1.000, respectively). ψεRACK induced hyperalgesia across groups (*P* < 0.001), and this was reduced by CP612 at all time points tested (Figure 3) [F _PKC_ _inhibition_ (1,10) = 78.74, *P* < 0.001; F _Time_ (6,60) = 19.87, *P* < 0.0001; F _Time__x__PKC_ _inhibition_ (6,60) = 7.29, *P* < 0.001]. Given the near-complete reversal, these experiments indicate that CP612 inhibits PKCε-dependent mechanical hyperalgesia.

### CP612 reduces paclitaxel-induced hyperalgesia.

**Figure 4:**
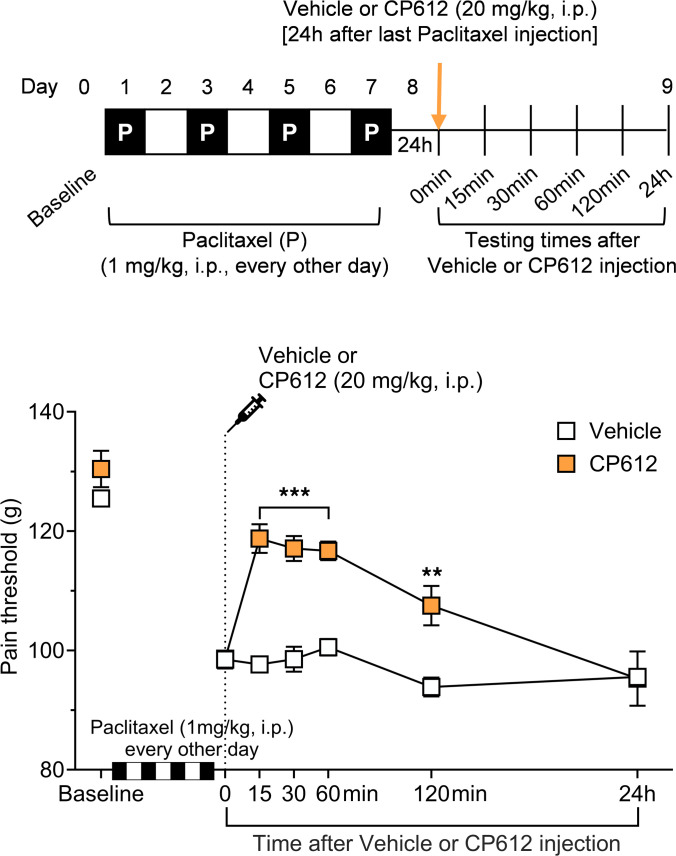
Hyperalgesia induced by the chemotherapeutic drug paclitaxel. Repeated administration of paclitaxel (1 mg/kg, i.p. every other day for a total of 4 injections) induced hyperalgesia in male rats. This was attenuated by CP612 (20 mg/kg, i.p.), up to 120 minutes after its administration. Data are shown as mean ± SEM (*n* = 6 paws per group). **P < 0.01, ***P < 0.001 compared with the same time points in the vehicle group using Tukey’s post hoc test.

Repeated administration of the cancer chemotherapeutic drug paclitaxel produces long-lasting mechanical hyperalgesia (18), due to CIPN. We tested if CP612 reverses this hyperalgesia. Male rats received repeated injections of paclitaxel (1 mg/kg, i.p. on days 1, 3, 5, and 7). Twenty-four hours after the last dose of paclitaxel, rats were administered vehicle (2 mL/kg, i.p.) or CP612 (20 mg/kg, i.p.). We measured nociceptive thresholds at baseline, 24 hours after the last injection of paclitaxel (time 0), and 15, 30, 60, and 120 minutes as well as 24 hours after administration of CP612 or vehicle (Figure 4). Pain thresholds at baseline and at time 0 did not differ significantly across groups (*P* = 0.947 and *P* = 1.000, respectively). Paclitaxel induced hyperalgesia across groups (*P* < 0.001), and this was attenuated 15, 30, 60, and 120 minutes after the administration of CP612 (Figure 4) [F _PKC_ _inhibition_ (1,10) = 33.74, *P* < 0.0002; F _Time_ (6, 60) = 59.35, *P* < 0.0001; F _Time__x__PKC_ _inhibition_ (6,60) = 10.19, *P* < 0.0001]. This decrease in hyperalgesia dissipated 1 day after the administration of CP612. These results indicate that CP612 blocks paclitaxel-induced hyperalgesia, an effect that is no longer present after 24 hours.

### CP612 prevents and reverses hyperalgesia induced by morphine withdrawal.

**Figure 5:**
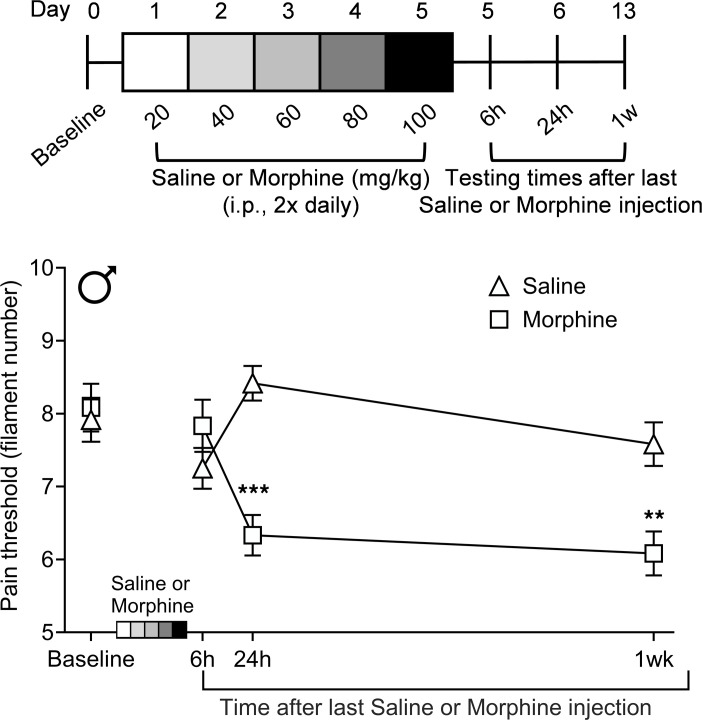
Time course of hyperalgesia induced by morphine withdrawal. After repeated administration of morphine (20–100 mg/kg, i.p.) twice daily for 5 days in male mice, hyperalgesia was not present at 6 hours but was present at 24 hours after the last injection of morphine and persisted for 1 week. No hyperalgesia developed after repeated administration of saline. Data are shown as mean ± SEM (*n* = 6 per group). **P < 0.01, ***P < 0.001 compared with the same time points in the group receiving repeated injections of saline using Tukey’s post hoc test.

Withdrawal from repeated administration of morphine produces hyperalgesia in rodents that is long lasting (19). To examine if CP612 alters this hyperalgesia, we first identified the time points at which hyperalgesia develops in male C57BL/6J mice. Mice received repeated injections of saline (10 mL/kg, i.p.) or morphine (20–100 mg/kg, i.p.) twice daily for 5 days, to induce dependence. Withdrawal from repeated administration of morphine induced hyperalgesia in a time-dependent manner (Figure 5) [F _Opioid_ _treatment_ (1,10) = 7.85, *P* < 0.02; F _Time_ (3,30) = 6.03, *P* < 0.003; F _Time__x__Opioid_ _treatment_ (3,30) = 10.67, *P* < 0.0001]. Specifically, hyperalgesia was not present at 6 hours but was present at 24 hours and 1 week after the last injection of morphine. Repeated administration of saline did not alter pain thresholds at any time tested.

**Figure 6:**
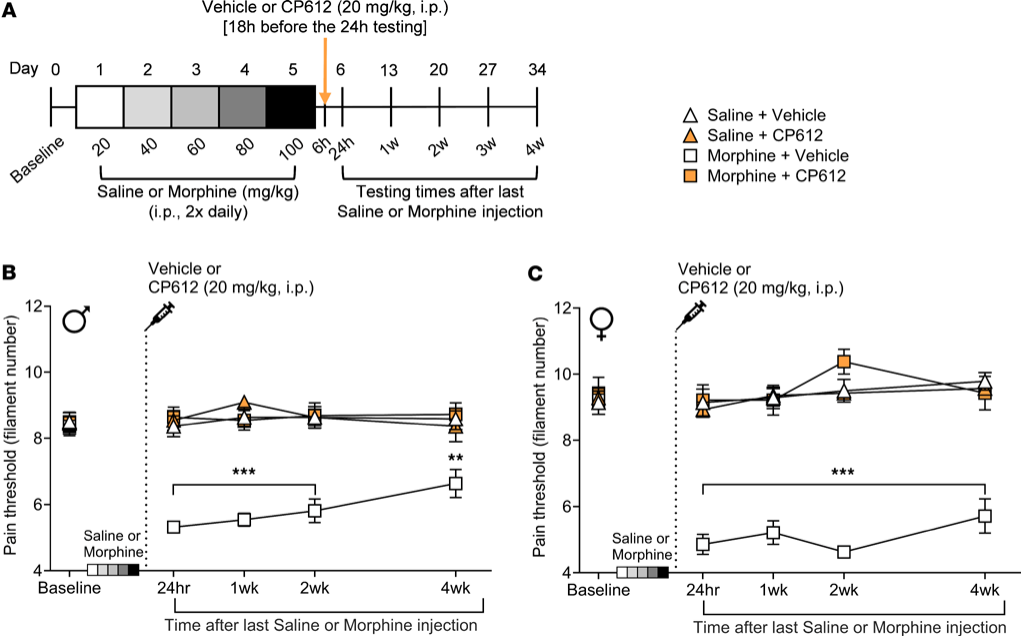
Prevention of hyperalgesia due to morphine withdrawal. (**A**) Experimental timeline. (**B** and **C**) Withdrawal from repeated administration of morphine (20–100 mg/kg, i.p.) induced hyperalgesia in male (**B**) and female (**C**) mice. This was prevented by CP612, up to 4 weeks after its administration. No hyperalgesia developed after repeated administration of saline. Data are shown as mean ± SEM (*n* = 11 for each male group, *n* = 7 for each female group). ***P < 0.001 and **P < 0.01 compared with the same time points in all other groups using Tukey’s post hoc test. For clarity, data from males and females are plotted separately.

We next investigated if CP612 prevents hyperalgesia induced by withdrawal from repeated administration of morphine, in male and female C57BL/6J mice. Mice received repeated injections of saline (10 mL/kg, i.p.) or morphine (20–100 mg/kg, i.p.) twice daily for 5 days. Six hours after the last injection (i.e., prior to the development of hyperalgesia), mice were administered vehicle (10 mL/kg, i.p.) or CP612 (20 mg/kg, i.p.). Baseline pain thresholds did not differ significantly before treatment with saline, morphine, vehicle, or CP612 and across sexes (all *P* > 0.99). Withdrawal from repeated administration of morphine produced hyperalgesia that was prevented by administration of CP612, whereas withdrawal from repeated administration of saline did not produce hyperalgesia and was not modified by administration of CP612; these effects did not differ across sexes (Figure 6, A–C) [F _Opioid_ _treatment_ (1,64) = 50.85, *P* < 0.001; F _PKC_ _inhibition_ (1,64) = 52.08, *P* < 0.001; F _Opioid_ _treatment__x__PKC_ _inhibition_ (1,64) = 51.50, *P* < 0.001; F _Time__=__20.43_, *P* < 0.001; F _Sex__x__Opioid_ _treatment__x__PKC_ _inhibition_ (1,64) = 2.34, *P* > 0.131; F _Time__x__Sex__x__Opioid_ _treatment__x__PKC_ _inhibition_ (4,256) = 1.23, *P* > 0.297]. These findings indicate that a single dose of the PKCε inhibitor can prevent hyperalgesia induced by opioid withdrawal.

**Figure 7:**
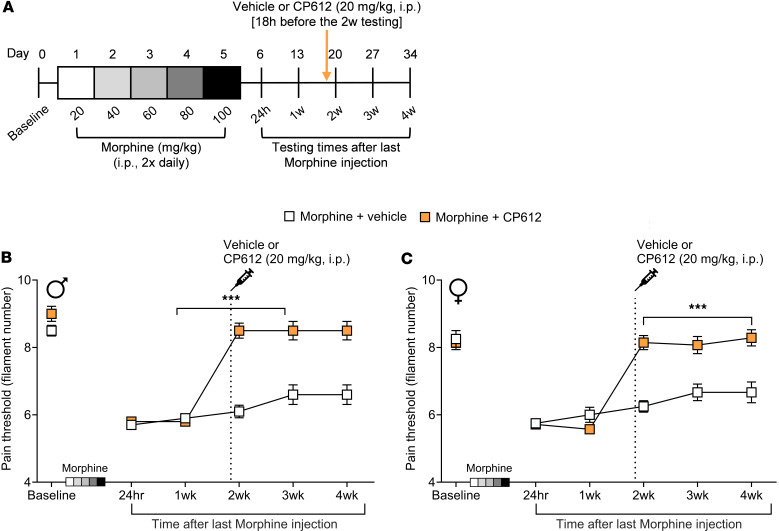
Reversal of hyperalgesia due to morphine withdrawal. (**A**) Experimental timeline. (**B** and **C**) Withdrawal from repeated administration of morphine (20–100 mg/kg, i.p.) induced hyperalgesia in male (**B**) and female (**C**) mice. This was reversed by CP612, administered 2 weeks after hyperalgesia was established and lasted for an additional 2 weeks. Data are shown as mean ± SEM (*n* = 5–7 per group). ***P < 0.001 compared with the same time points in the vehicle group using Tukey’s post hoc test. For clarity, data from males and females are plotted separately.

We next tested if CP612 reverses hyperalgesia induced by withdrawal from repeated administration of morphine in male and female C57BL/6J mice. Since the pain thresholds of mice receiving repeated administration of saline were not affected by treatment with vehicle or CP612 injection (Figure 6), mice in this experiment were only treated with morphine. Male and female C57BL/6J mice received repeated injections of morphine (20–100 mg/kg, i.p.) twice daily for 5 days. Two weeks after the last injection of morphine (i.e., after the development of hyperalgesia), mice were administered vehicle (10 mL/kg, i.p.) or CP612 (20 mg/kg, i.p.). Baseline pain thresholds did not differ significantly before repeated administration of morphine and across sexes (all *P* > 0.373). Hyperalgesia was apparent at 24 hours and 1 week after the last injection of morphine (Figure 7, A–C) [F _time_ (5,95) = 0.120.51 *P* < 0.001]; CP612 reversed this hyperalgesia (Figure 7, A–C) [F _PKC_ _inhibition_ (1,19) = 58.52, *P* < 0.001], and this occurred similarly across sexes [F _sex__x__PKC_ _inhibition_ (1,19) = 2.66, *P* > 0.119] and time [F _time__x__sex__x__PKC_ _inhibition_ (5,95) = 0.20, *P* > 0.960]. Both male and female mice that received vehicle continued to show hyperalgesia up to 4 weeks after the last injection of morphine. In contrast, mice that received CP612 showed a persistent reversal of hyperalgesia. These results indicate that a single dose of the PKCε inhibitor can reverse hyperalgesia induced by opioid withdrawal.

### CP612 has a low addictive potential.

We tested the addictive potential of CP612 by examining the extent to which rats are willing to work to obtain CP612. Rats were initially trained to self-administer either saline, morphine (500 μg/kg/i.v. infusion), or 1 of 3 different doses of CP612 (75, 150, or 300 μg/kg/i.v. infusion) for 4 days at fixed ratio 1 (FR1, where 1 nose poke delivers 1 infusion). During this phase, rats learned self-administration behavior, as attested by greater responding in the active hole than the inactive hole [F _hole_ _type_ (1,37) = 129.11, *P* < 0.001, data not shown]. Intake differed across different treatment groups [F _Group_ (4,37) = 8.50, *P* < 0.01] in that the group self-administering morphine took fewer infusions (average intake, 9.1 infusions) compared with all other groups, which had similar levels of intake (average intake, 28.0, 29.3, 30.8, and 29.9 for rats self-administering saline or CP612 at 75, 150, or 300 μg/kg/i.v. infusion, respectively).

**Figure 8:**
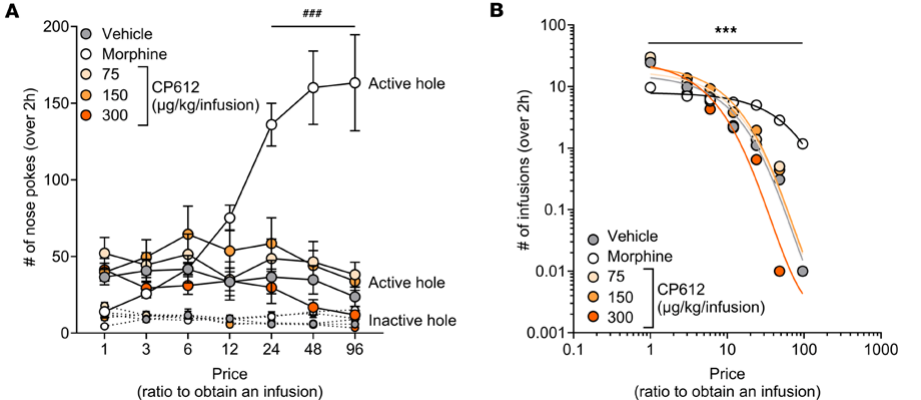
Self-administration of CP612. (**A**) Increasing the ratio to obtain an infusion across self-administration sessions produced a concomitant increase in responding in the active hole in rats self-administering morphine (500 μg/kg/i.v. infusion) but not in rats self-administering vehicle or different doses of CP612 (75, 150, and 300 μg/kg/i.v. infusion). (**B**) Rats self-administering morphine had a lower elasticity value (α = 0.4 × 10^3^) compared with all other groups (α = 1.1, 0.9, 0.9, 1.2 × 10^3^ for vehicle and CP612 at 75, 150, and 300 μg/kg/i.v. infusion). They also had a higher price value to switch from inelastic to elastic behavior (*P*_max_ = 50.1) compared with all other groups (*P*_max_ = 4.1, 6.5, 6.1, 2.5 for vehicle and 75, 150, and 300 μg/kg/i.v. infusion). Data are shown as mean ± SEM (*n* = 10 for vehicle, *n* = 9 for morphine, *n* = 7–8 for each CP612 group). ^###^*P* < 0.001 compared with their infusions at FR1 and the same ratios between rats self-administering morphine and all other groups using Tukey’s post hoc test. ***P < 0.001 for *P*_max_ and ^††^*P* < 0.01 for α, between rats self-administering morphine compared with all other groups using Tukey’s post hoc test. AH, active hole; IH, inactive hole.

During the second phase, the ratio to obtain an infusion increased geometrically every other day (FR1, FR3, FR6, FR12, FR24, FR48, and FR96). This increase in ratio produced an increase in responding in the active hole (Figure 8A) [F _Ratio_ (6,222) = 7.75, *P* < 0.001], and this occurred differentially across groups [F _Ratio__x__Group_ (24,222) = 15.71, *P* < 0.001]. Thus, rats self-administering saline or any dose of CP612 did not significantly increase responding in the active hole when the ratio to obtain an infusion increased, whereas rats self-administering morphine showed increased responding with the increase in ratio (Figure 8A). There were no group differences in responding in the inactive hole [F _Group_ (4,37) = 1.40, *P* > 0.25].

The increase in ratio also changed the number of self-infusions [F _Ratio_ (6,222) = 171.68, *P* < 0.001], and this occurred differentially across groups [F _Ratio__x__Group_ (24,222) = 6.49, *P* < 0.001]. Thus, rats self-administering saline or any dose of CP612 showed a similar decrease in the number of self-infusions when the ratio to obtain an infusion increased. In contrast, rats self-administering morphine did not significantly decrease the number of self-infusions until FR98.

Data (number of self-infusions) were also fitted in an economic-demand curve, which provides an estimate of the motivation to obtain drugs and their addictive potential (20). We measured elasticity (α), which is the relative change in consumption as a function of price (i.e., ratio of nose pokes required to obtain an infusion). Behavior is considered “inelastic” when consumption is insensitive to price and “elastic” when consumption is sensitive to price. We also measured *P*_max_, which is the price at which intake switches from inelastic to elastic. Rats self-administering morphine had a lower elasticity (α) compared with all other groups [F _Group_ (4,37) = 5.12, *P* < 0.01] and had a greater *P*_max_ [F _Group_ (4,37) = 18.67, *P* < 0.001] (Figure 8B). Taken together, these findings indicate that CP612 is highly unlikely to be addictive.

### CP612 does not alter morphine self-administration.

**Figure 9:**
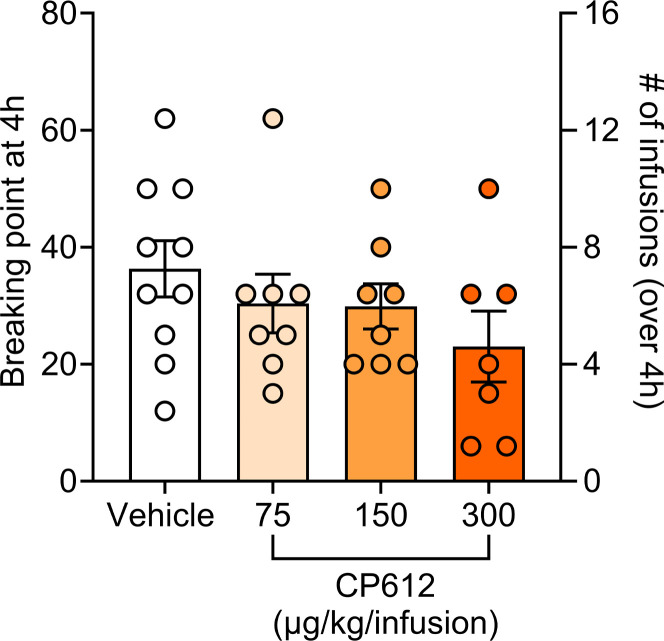
Self-administration of morphine when adding CP612 to the morphine solution. Adding CP612 (75, 150, and 300 μg/kg/i.v. infusion) to the morphine solution (500 μg/kg/i.v. infusion) during self-administration did not modify responding in a progressive ratio test, where the ratio to obtain an infusion was increased progressively within a self-administration session. This was measured as breaking point (highest ratio reached to earn an infusion of drug) and number of self-infusions. Data are shown as mean ± SEM; dots are scores from individual rats (*n* = 7–10 per group).

Because many patients with chronic pain take opioids for pain management (8, 9), we wanted to know if CP612 would change opioid intake. Therefore, we investigated if adding CP612 to the morphine solution during self-administration (i.e., a cocktail of morphine and CP612) modifies self-administration of morphine. We tested rats from the prior experiment for self-administration of morphine (500 μg/kg/i.v. infusion). Rats were first tested under a FR6 to reestablish responding. Then, we added vehicle or 3 different doses of CP612 (75, 150, or 300 μg/kg/i.v. infusion) to the morphine solution. We examined its effects during a 4-hour session using a within-session progressive ratio schedule of reinforcement, in which the ratio to obtain an infusion is increased semilogarithmically within a self-administration session as 1, 2, 4, 6, 9, 12, 15, 20, 25, etc., thereby allowing testing over a single day (21). At all tested doses, CP612 did not modify responding, measured as the number of infusions and breaking point (i.e., the highest ratio rats complete to earn an infusion of drug) (Figure 9) [F _PKC_ _inhibition_ (3,29) = 1.67, *P* > 0.195 and = 1.21, *P* > 0.324 for infusions and breaking point, respectively].

**Figure 10:**
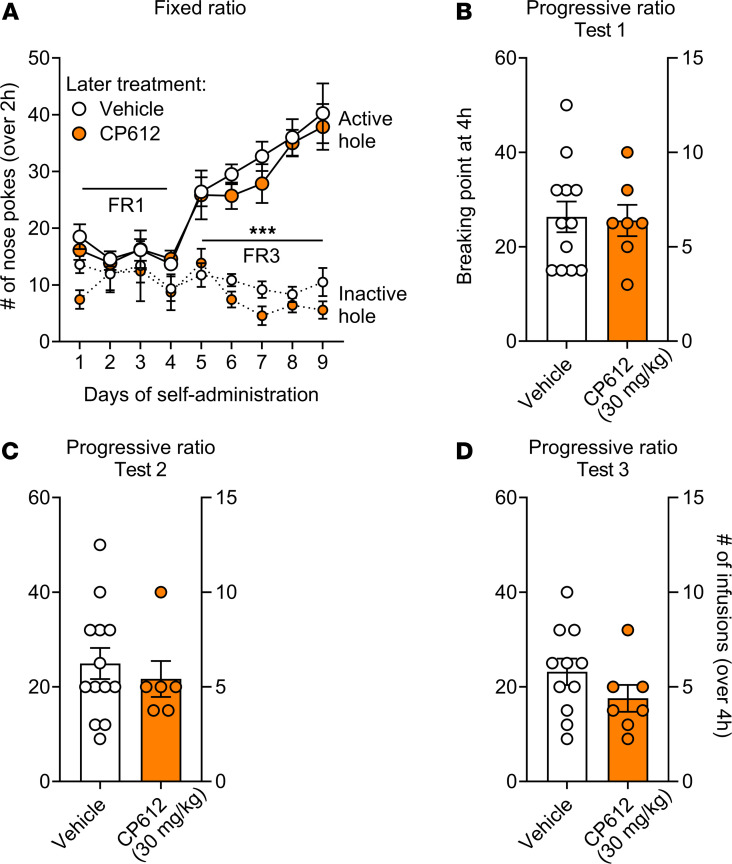
Self-administration of morphine after administering CP612 six hours beforehand. (**A**) Rats acquired morphine self-administration behavior at FR1, and this responding was increased when the ratio to obtain morphine was increased to FR3. This occurred to the same extent in rats that would later receive vehicle or CP612. AH, active hole; IH, inactive hole. During the progressive ratio tests, the ratio to obtain an infusion was increased progressively within a self-administration session. Administration of CP612 (30 mg/kg, i.p.) 6 hours beforehand did not modify morphine intake or responding. (**B**–**D**) This was measured as breaking point (highest ratio reached to earn an infusion of drug) and number of self-infusions the day after the last self-administration session (**B**), after 1 day of withdrawal (**C**), or after the administration of naloxone (**D**) (0.03 mg/kg, s.c.). Data are shown as mean ± SEM; dots are scores from individual rats (*n* = 6–13 per group). ***P < 0.001 compared with the inactive hole using Tukey’s post hoc test.

We next investigated if CP612, administered prior to a self-administration session, modifies self-administration of morphine. A separate group of rats was first trained to self-administer morphine (500 μg/kg/i.v. infusion) at FR1 and then at FR3. Rats learned self-administration behavior, as attested by greater responding in the active hole versus the inactive hole [F _Hole_ _type_ (1,17) = 173.96, *P* < 0.001]; this occurred to a similar extent in rats that would later receive vehicle or CP612 (Figure 10A) [F _Hole_ _type__x__PKC_ _inhibition_ (1,17) = 0.08, *P* > 0.78]. The average intake of morphine was 9.26 infusions, and this intake was similar in rats that would later receive vehicle or CP612 [F _PKC_ _inhibition_ (1,17) = 0.02, *P* > 0.89; F _Days__x__PKC_ _inhibition_ (8,126) = 0.65, *P* > 0.73].

CP612 (30 mg/kg, i.p.), administered 6 hours prior, did not modify morphine intake or responding, measured as the number of morphine infusions and breaking point; this was true for all successive progressive ratio tests: test 1 was done the day after initial self-administration (Figure 10B) [F _PKC_ _inhibition_ (1,17) = 0.01, *P* > 0.94 and = 0.02, *P* > 0.88 for infusions and breaking point, respectively], test 2 was done after 1 day of withdrawal from self-administration (Figure 10C) [F _PKC_ _inhibition_ (1,17) = 0.16, *P* > 0.69 and = 0.34, *P* > 0.56 for infusions and breaking point, respectively], and test 3 was done after the administration of naloxone (0.03 mg/kg, s.c.) (Figure 10D) [F _PKC_ _inhibition_ (1,17) = 1.66, *P* > 0.219 and = 1.80, *P* > 0.19 for infusions and breaking point, respectively]. Similar results were obtained when CP612 was administered 18 hours prior to the self-administration session (Supplemental Figure 1).

### CP612 reduces somatic signs of morphine withdrawal but not conditioned place aversion.

**Figure 11:**
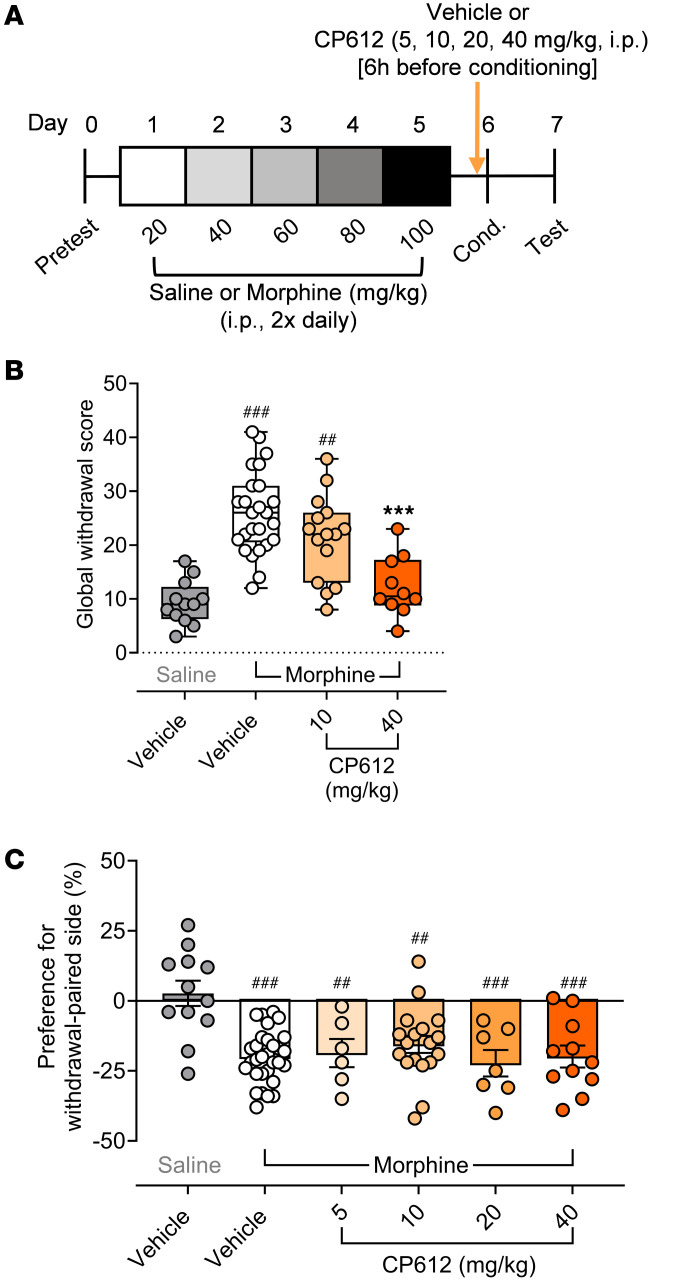
Morphine withdrawal and conditioned place aversion (CPA) precipitated by naloxone. (**A**) Experimental timeline. (**B**) On conditioning day, naloxone (5 mg/kg, i.p.) evoked signs of withdrawal in male mice that received repeated administration of morphine (20–100 mg/kg, i.p.) and were treated with vehicle (*n* = 25) or a low dose of CP612 (10 mg/kg, i.p., *n* = 15), but this was decreased in mice treated with a high dose of CP612 (40 mg/kg, i.p., *n* = 10). (**C**) On test day, prior administration of naloxone produced CPA in mice that received repeated administration of morphine, and this occurred similarly in mice that were treated with vehicle (*n* = 30) or different doses of CP612 (5, 10, 20, 40 mg/kg, i.p., *n* = 6, 18, 7, 11, respectively). For **B**, box and whiskers are median and 25% interquartile intervals; dots are scores from individual mice. For **C**, Data are shown as mean ± SEM; dots are scores from individual mice. ^##^*P* < 0.01 and ^###^*P* < 0.001 compared with Saline + Vehicle: ***P < 0.001 compared with Morphine + Vehicle using Tukey’s post hoc test.

In addition to hyperalgesia, withdrawal from repeated administration of morphine produces somatic signs of withdrawal and conditioned place aversion. We tested the effects of CP612 on these responses. A pilot experiment using C57BL/6 mice showed poor conditioned place aversion, so we used DBA/2J mice for these experiments, since they show more robust conditioned place aversion (22). Male DBA/2J mice received repeated injections of saline (10 mL/kg, i.p.) or morphine (20–100 mg/kg, i.p.) twice daily for 5 days. On day 6 (the conditioning day), mice were pretreated with vehicle (10 mL/kg, i.p.) or CP612 (different doses, i.p.), followed by a single injection of saline (10 mL/kg, i.p.) or morphine (100 mg/kg, i.p.) 4 hours later. Two hours after this last injection of saline or morphine, mice were administered naloxone (5 mg/kg, i.p.) to precipitate somatic signs of withdrawal and conditioned place aversion. Withdrawal from repeated administration of morphine evoked somatic signs of withdrawal (Figure 11, A–C, and Supplemental Figure 2); only the highest dose of CP612 (40 mg/kg) reduced them (Figure 11, A–C) [H _PKC_ _inhibition_ (3, *n* = 62) = 33.34, *P* < 0.001]. Withdrawal from repeated administration of morphine also evoked conditioned place aversion (Figure 11, A and C) [F _PKC_ _inhibition_ (5,78) = 7.16, *P* < 0.001; mice receiving repeated administration of saline with vehicle versus mice receiving repeated administration of morphine with vehicle, *P* < 0.001], and this was not modified by CP612 at any dose tested (all *P* > 0.079).

## Discussion

We describe a small molecule inhibitor of PKCε, CP612, that reduces hyperalgesia evoked by activation of PKCε in peripheral nociceptors and by administration of the chemotherapeutic drug paclitaxel in rats. CP612 itself had no addictive potential, nor did it modify the addictive potential of morphine but it prevented and reversed hyperalgesia induced by morphine withdrawal. This effect did not generalize well to other features of opioid withdrawal such as conditioned place aversion or somatic signs of withdrawal, which was reduced only at a high dose but not at a low dose of CP612 that inhibited pain.

In vitro analysis showed that CP612 inhibited PKCε competitively with respect to ATP with low nanomolar potency. CP612 interacted only with ROCK1 and ROCK2, PKC isozymes most related to PKCε (PKCδ, PKCη, PKCθ), and 5 other kinases when screened at 200 nM against a panel of 468 native kinases. CP612 was 40-fold more potent at inhibiting PKCε than ROCK1 and, within the novel PKC subfamily, was most potent against PKCε. Pharmacokinetics studies in rats showed that CP612 was brain penetrant and had a long CNS half-life when administered by i.p. or i.v. injection. Together, these properties suggest that CP612 is a potent and relatively selective inhibitor of PKCε that has favorable CNS drug-like physical-chemical properties based on those measured for clinically useful CNS drugs by CNS multiparameter optimization analysis (17).

Paclitaxel-induced hyperalgesia is long lasting in rats, persisting for up to 2 weeks after the last injection (18). Although CP612 reduced hyperalgesia in this model, the effect dissipated within 24 hours. We also found that hyperalgesia produced by withdrawal from repeated administration of morphine persisted in mice for over 1 week, as reported by others (19, 23, 24). CP612 abolished this hyperalgesia when administered before it had developed and later when it was well established. Unlike with paclitaxel-induced hyperalgesia, this effect of CP612 was persistent, lasting several weeks. These differences in response may reflect differences in pathophysiology between paclitaxel, which causes a toxic neuropathy, and morphine, which evokes a neuroadaptive response. That the effect of a single injection of CP612 was long lasting suggests that a single dose of a PKCε inhibitor could be useful to prevent or reverse hyperalgesia provoked by opioid withdrawal.

It is difficult to determine from our study if CP612 decreased hyperalgesia through central or peripheral mechanisms. Hyperalgesia after paclitaxel is mostly peripheral at the time points tested (25, 26), suggesting that the action of CP612 was peripheral. On the other hand, hyperalgesia after withdrawal from opioids involves central mechanisms (27), suggesting that CP612 also acts centrally. Further studies are needed to pinpoint where CP612 acts to reduce hyperalgesia in these different models of pain.

Previous studies have identified sexual dimorphism for PKCε in different models of chronic pain. Inhibition of PKCε was only able to attenuate hyperalgesia in male but not female rats with CIPN induced by vincristine or diabetic hyperalgesia induced by streptozotocin (28, 29). We found no sex differences in the effect of CP612 on prevention or reversal of hyperalgesia induced by opioid withdrawal. However, further work is needed to determine if there are sex differences in the effects of CP612 in paclitaxel-induced hyperalgesia since studies to date have only investigated the role of PKCε in male rats (18) and mice (30).

When developing new pain medications, it is important to determine if they have rewarding properties themselves, thereby posing an addiction risk. We tested this possibility by comparing self-administration of CP612 to self-administration of morphine or saline. This was done using a between-session progressive ratio test, whereby the ratio to obtain the drug increases geometrically across successive days. This allows fitting data to an economic demand curve, that can estimate the motivation to obtain drugs and their “addictive potential” (20). Rats were initially tested at a fixed ratio 1 (where 1 nose-poke yields 1 infusion of drug). During this phase, self-administration of morphine was low, as is expected at this dose (500 μg/kg/i.v. infusion) (31, 32). Instead, self-administration of saline was higher and likely occurred because nose-poking in the active hole was coupled with the presentation of a light cue, which prompted responding. Self-administration of saline occurred to the same extent as any dose of CP612 tested. As the ratio to obtain infusions increased, responding increased for morphine but not for saline or any dose of CP612. In fact, the maximum price rats were willing to pay for an infusion of drug was highest for morphine compared with all other groups, which did not differ from each other. Furthermore, intake of morphine was “inelastic” (did not change as the price to obtain the drug increased) compared with intake of saline and any dose of CP612, which was “elastic” (decreased as the price to obtain the drug increased). These results indicate that CP612 is unlikely to be addictive.

Because nonopioid pain medications might be taken with opioids, we tested if adding CP612 to the morphine solution during self-administration could enhance self-administration of morphine. In a progressive ratio test, CP612 did not modify morphine intake at any dose tested, suggesting that CP612 is unlikely to increase motivation to self-administer morphine. We also tested if administering CP612 to rats that already learned to self-administer morphine could modify self-administration of morphine. Again, CP612 had no effect on morphine self-administration. These results suggest that CP612 is unlikely to modify morphine self-administration. A caveat of these experiments is that rats were trained to self-administer morphine at 500 μg/kg/i.v. infusion. This dose was chosen because it produces i.v. intake of approximately 5–7.5 mg/kg, which is within the range of i.v. doses that produce analgesia in rats (33). However, this dose does not produce dependence. It is therefore possible that, had rats self-administered higher doses of morphine leading to dependence, we might have observed different effects.

In conclusion, we have identified a nonaddictive, small molecule inhibitor of PKCε, CP612, that was effective in treating pain in a model of CIPN. CP612 had the added and important benefit of preventing and reversing hyperalgesia evoked by opioid withdrawal. Long-term opioid therapy can become ineffective in controlling pain and improving function, and because opioid tapering can worsen pain, reducing the dose of opioid medications can be extremely difficult (7). Because CP612 was effective in preventing and reversing hyperalgesia due to opioid withdrawal, PKCε inhibitors may provide a new therapeutic option for individuals who are discontinuing opioid treatment and find it difficult to become opioid-free.

## Methods

*Sex as a biological variable*. Ours studies examined male and female rats and mice unless specified; similar finding were found in both sexes.

*Animals*. Rats (Sprague-Dawley) were acquired from Envigo and Charles River Laboratories and were housed 2–3 per cage. Upon arrival, male and female rats were 60–70 days old and weighed 250–275 g (males) and 175–199 g (females).

C57BL/6J and DBA/2J mice were acquired from The Jackson Laboratory and housed 2–3 per cage. Upon arrival, male and female mice were 8–9 weeks old and weighed 24–27 g (males) or 19–22 g (females).

Animals were housed on a 12:12 light/dark cycle with ad libitum access to water and laboratory chow (LabDiet). Experiments were performed 8–10 hours after lights on, except for conditioned place aversion and self-administration experiments, which were performed 3–6 hours after lights off, to facilitate conditioning and self-administration (34). All experiments were performed on separate groups of animals unless specified.

*Randomization and blinding*. For all experiments, animals within the same cage received the same treatment. In experiments where treatments were administered prior to obtaining outcome measures, animals were randomized in different experimental treatments based on their position on the housing rack, to ensure that treatments were similarly distributed based on row and position within the row. In experiments where treatments were administered after obtaining outcome measures, animals were distributed in the different treatment groups to ensure that each treatment group contained a representative sample of the population, based on the outcome measure.

For all studies where outcomes depended on subjective measures (pain thresholds and withdrawal scores), the experimenters were blind to the treatment conditions of the animals.

*Drugs and reagents*. Morphine sulfate and naloxone hydrochloride were obtained from Spectrum Chemical and diluted in saline (0.9% NaCl); doses of morphine are expressed as morphine base. Mepivacaine was obtained from TCI America and diluted in saline (0.9% NaCl). Paclitaxel was obtained from Sigma-Aldrich and dissolved in absolute ethanol and polyethoxylated castor oil (Cremophor EL; 1:1; Sigma-Aldrich) and further diluted in saline to a final concentration of 10% Cremophor EL. Sodium brevital was obtained from Henry Schein and was diluted in saline (0.9% NaCl). Lidocaine was obtained from Aspen Veterinary Resources and diluted in saline (0.9% NaCl). The ψεRACK peptide (HDAPIGYD) was obtained from AnaSpec Inc. CP612 was synthesized in enantiomerically enriched form (>98% enriched) by Stanton McHardy (University of Texas San Antonio, San Antonio, Texas, USA) and diluted in a vehicle solution made of 5% Tween 80 (Sigma-Aldrich) and saline (0.9% NaCl) for i.p. injections and in saline (0.9% NaCl) for i.v. infusions.

*Kinetic aqueous solubility*. Test compounds were diluted 100-fold from 1 mM DMSO stocks into 0.1M phosphate-buffered saline (PBS) in triplicate. Diluted samples were shaken at room temperature at 600 rpm for 3 hours and then filtered by centrifugation (0.2 μm, PVDF) at 1,000*g* for 1 minute. Aliquots of 100 μL were diluted into 3 volumes of acetonitrile for extraction, vortexed for 15 seconds, centrifuged at 16,000*g* for 10 minutes at 4°C, and then analyzed by liquid chromatography–mass spectrometry (LC-MS).

*LogD determination*. Test compounds were diluted by adding 12 μL of 1 mM DMSO stocks to 1,188 μL of 1:1 0.1M PBS/n-octanol in duplicate. Samples were vortexed, shaken at 600 rpm for 3 hours at 37°C. Samples (100 μL) of each phase layer were extracted in 3 volumes of acetonitrile for 15 minutes, centrifuged at 10,000*g* for 10 minutes, and then analyzed by LC-MS. The ratio of concentrations measured in the organic and aqueous phases was log_10_ transformed to determine the logD.

*Plasma protein binding*. Binding of CP612 to human plasma proteins was measured by equilibrium dialysis using a Rapid Equilibrium Dialysis (RED) Device (Thermo Fisher Scientific). The device uses plates with wells preloaded with equilibrium dialysis membrane inserts, each of which includes 2 side-by-side chambers separated by a dialysis membrane. Testosterone was used as a control for high protein binding, and metoprolol was used as a control for low protein binding. All compounds were tested in triplicate. In total, 7 μL of each test compound (1 mM in DMSO) were added to 693 μL of human plasma (BioIVT). A total of 200 μL of diluted test compound was then added to the plasma chambers of triplicate wells of a 48-sample RED plate (Thermo Fisher Scientific). In total, 400 μL of 0.1M PBS buffer were added to each buffer chamber. The plate was sealed with adhesive foil and incubated at 37°C with shaking for 4 hours, after which 30 μL were removed from each chamber and diluted with either 30 μL of plasma (for buffer chamber samples) or 30 μL of 0.1M PBS (for plasma chamber samples). Samples were extracted with 3 volumes of acetonitrile, centrifuged at 16,000*g* at 4°C for 10 minutes, and analyzed by LC-MS to calculate the amount of drug in each chamber. The percentage of free compound was calculated as the concentration in the buffer chamber/concentration in the plasma chamber × 100%. The percentage of protein-bound compound was calculated as 100% – the percentage of free compound.

*Plasma stability*. To determine in vitro plasma stability, we calculated in vitro plasma half-life. We incubated test substances (0.6 μL) with 60 μL human plasma (BioIVT) in quadruplicate at 37°C. Procaine was tested as a positive control and procainamide as a negative control. Samples were incubated with continuous shaking at 600 rpm for 0, 15, 30, 60, or 180 minutes. Reactions were quenched with 3 volumes of acetonitrile, extracted for 10 minutes, centrifuged at 2,350*g* for 20 minutes at 4°C, and then analyzed by LC-MS to determine first-order elimination rate constants k and values for drug half-life and clearance for each compound.

*Microsomal clearance*. To determine if CP612 was a likely substrate for first-pass hepatic metabolism, we measured the stability of CP612 in the presence of human liver microsomes (BioIVT) pooled from over 100 male and female individuals. Testosterone was tested as a positive control. Test compounds were diluted to 10 μM in 0.1M PBS, and then 51 μL were dispensed in quadruplicate into wells of a 96-well plate, which was shaken at 600 rpm at 37°C. Reactions were initiated by adding 1.5 μL of microsomes (20 mg/mL) to the wells and 3 minutes later adding 7.5 μL of 8 mM NADPH (1 mM final concentration). Samples incubated without NADPH were used as negative controls. Reactions proceeded for 0, 5, 15, 30, or 60 minutes and were quenched with 3 volumes of acetonitrile, extracted for 10 minutes, centrifuged 2,350*g* for 20 minutes at 4°C, and analyzed by LC-MS to determine first-order elimination rate constants and values for drug half-life and clearance for each compound.

*Kinase assays*. CP612 was assayed against His-AVI–tagged human PKCε (1 nM) produced in Sf9 cells, and ROCK1 (Carna Biosciences; 5 nM) using the Lance-FRET kinase assay system (PerkinElmer Revvity). Other human recombinant PKCs (SignalChem Biotech) were assayed in triplicate at the following concentrations: PKCβII 1.6 nM, PKCγ 500 pM, PKCδ 5 nM, PKCθ 500 pM, and PKCζ 500 pM. PKCs were incubated in a 15 μL reaction volume with kinase buffer (20 mM Tris-HCL [pH 7.4], 10 mM MgCl_2_, 0.25 mM EGTA, 0.1 mg/mL BSA, and 0.15% Triton X-100), 50 nM ULight-labeled CREBtide CKRREILSRRPSYRK (PerkinElmer, TRF0107, Revvity), and test compound. Reactions were initiated by adding 2.5 μM ATP at 27°C and were terminated after 60 minutes with 10 μL of 2.5× Stop Solution/Detection Mix containing 20 mM EDTA and 4 nM LANCE Ultra Europium anti–phospho-PKC (Ala25Ser) peptide antibody (PerkinElmer, TRF0200, Revvity). After incubation at 27°C for another 60 minutes, phosphorylation was detected using a FlexStation 3 Microplate Reader (Molecular Devices) in LANCE TR-FRET mode (excitation = 340 nm, emission = 665 nm) and expressed as relative fluorescence units (RFU). The percentage of inhibition was calculated as: (signal without test compound − signal with test compound)/(signal without test compound − signal without ATP) × 100. The Z’ factor for this assay ranged between 0.72 and 0.86.

*Pharmacokinetic studies in rats*. Pharmacokinetics studies were contracted to the Drug Metabolism and Pharmacokinetics Core at Scripps Research. CP612 was dissolved at in 10% Tween-80/90% water and administered at 40 mg/kg/5 mL. Six rats were injected i.v. and 6 were injected i.p. Three rats were used to collect a full plasma time course from 5 minutes to 24 hours after dose. Then, brains were collected from these rats at 24 hours and from 3 additional rats at 2 hours after administration of compound. One rat receiving i.p. administration of CP612 was excluded as it had low/nondetectable levels indicating missed i.p. injection in that rat. Plasma samples were diluted 10-fold in blank plasma. Each brain was disrupted in potassium phosphate buffer (pH 7.4; 9 μL per 1mg tissue) on ice using brief sonication pulses. CP612 was detected with an API Triple Quad 5500 LC-MS/MS (SCIEX) using an MRM(+) method: CP612 (463/282, *m/z*) with carbamazepine (238/195.1, *m/z*) as an internal standard.

### Mechanical nociception

#### Rats.

Mechanical nociceptive thresholds in rats were quantified by the Randall-Selitto paw pressure test using an Ugo Basile Analgesy-Meter. This device applies a linearly increasing mechanical force to the dorsum or plantar region of the rat hind paw. The mechanical nociceptive threshold is defined as the force in grams at which a rat withdraws its paw. The higher the force to elicit paw withdrawal, the higher the pain threshold (and the lower the sensitivity to pain). For studies testing nociceptive thresholds after the PKCε activator ψεRACK, or after the cancer chemotherapeutic drug paclitaxel, rats were placed into cylindrical acrylic restrainers for 40 minutes before starting each session. The restrainers had lateral ports to allow access to the hind paw, as previously described (35), and pain thresholds were measured by applying force to the dorsum of the hind paw. Prior to starting experiments, baseline mechanical nociceptive threshold was obtained as the mean of 2–3 readings on 1 hind paw in each rat. Rats were then distributed in each experimental group according to their baseline level of response, to ensure similar baseline values across groups.

#### Mice.

Mechanical nociceptive thresholds in mice were quantified using the simplified up-down (SUDO) method with von Frey filaments (36). The testing apparatus consisted of an elevated platform (34 × 72.5 × 35 in cm) with a metal mesh floor and square plastic compartments (10 × 14 × 10 in cm) on top to separate each mouse. von Frey filaments (Semmes-Weinstein monofilaments; Stoelting) were pressed against the center of the plantar surface of the hind paw until they buckled and were held for a maximum of 5 seconds. The force to bend the filaments ranged from 0.008 g (filament 1) to 6 g (filament 12). Mice were acclimated to the test room and apparatus for 1 hour prior to testing. We started testing mice with filament 5 (bending force 0.16 g) and stepped up or down according to the response of the mouse. The filament that elicited a definite paw withdrawal or notable flinch was recorded. All measures were averaged between both hind paws and are expressed as filament number, which allows for parametric analysis (36). The higher the filament number, the higher the pain threshold (and the lower the sensitivity to pain). Prior to starting experiments, baseline threshold levels were obtained 3–4 times at 2- to 5-day intervals on each of the hind paws of the mouse. Mice were then distributed in each experimental group according to their baseline level of response, to ensure similar baseline values across groups.

### I.v. self-administration

#### Implantation of intravenous catheters.

Rats were anesthetized to implant catheters for intravenous self-administration. Anesthesia was induced by placing rats into an induction chamber filled with 5% isoflurane gas and was maintained with a vaporizer by delivering 2%–3% isoflurane through a nose cone (E-Z Anesthesia). To ensure sufficient anesthesia, breathing rate, pinch response, and body temperature were monitored throughout surgery, and anesthesia was adjusted when necessary. Areas around incisions were shaved with electric clippers (Andis Company) and were cleaned with 70% alcohol, followed by 10% betadine and Lanacaine (20% benzocaine, 0.2% benzethonium chloride, 36% ethanol). Incision sites were then infused with the local anesthetics mepivacaine (2%) for the back incision and lidocaine (0.5%) for the front incision. Silastic catheters were implanted in the right external jugular vein and passed under the skin to exit in the midscapular region. The catheters were accessible through a backport that was mounted onto a pedestal secured under the skin with surgical staples (Braintree Scientific Inc.). At the conclusion of each surgery, wounds were covered with topical triple antibiotic ointment (bacitracin, neomycin sulfate, polymyxin B sulfate; Medique Products). Systemic NSAID analgesics, either carprofen (Animal Health International, 5 mg/kg/mL, s.c.) or flunixin meglumine (Henry Schein, 2.5 mg/kg/0.5 mL, s.c.), were administered the day of surgery and 1–2 days following. Systemic antibiotic cefazolin (Henry Schein, 50 mg/kg/0.5 mL, i.v.) was administered the day of surgery and 2–6 days following, then reduced to 30 mg/kg/0.5 mL for the remainder of the experiment.

#### General procedures.

Self-administration procedures took place in oversized Med Associates operant chambers (41 × 24 cm floor area, 21 cm high) outfitted with photobeams to track locomotion and 2 nose holes to track responding (nose poking). Rats were placed daily in the operant chamber for 2–4 hours (depending on the study). The backport of the catheter was connected with a tether to an infusion pump (Med Associates). The tether was made of Tygon tubing (Cole Parmer) shielded by a metal spring (Instech Laboratories) to prevent rats from chewing on it. We also added a “nylabone” in the chamber for chewing, as rats self-administering morphine often chewed on their tethers. Nose-poking into 1 hole (“active hole”) activated the infusion pump to deliver an i.v. infusion of drug at the rate of 11–12 μL/s and a volume of 150 μL/kg, concomitant with a 15-second light cue within the hole and a 15-second time-out period. Nose poking into the other hole (“inactive hole”) had no consequences and was used to track non-goal-directed nose poking. We recorded the number of nose pokes and infusions using MED-PC IV software (Med Associates). At the end of each experiment, we tested catheter patency by administering the fast-acting anesthetic sodium brevital (5 mg/kg/0.5 mL, i.v.). Rats not immediately anesthetized were eliminated from the study. This excluded 2 rats from all experiments. For tests involving administration of vehicle or CP612 after acquisition of self-administration of morphine, rats were distributed in each experimental group according to their baseline intake of morphine, to ensure similar baseline values across groups.

#### Progressive ratio testing (between-session).

Rats were randomly assigned to different groups that were tested for self-administration of saline, morphine (500 μg/kg/i.v. infusion), or 3 doses of CP612 (75 or 150 or 300 μg/kg/i.v. infusion) for 2 hours per day under fixed ratio 1 (FR1, where 1 nose poke delivers 1 infusion) until behavior (number of infusions) was stable (varied less than 25% for 3 consecutive days); this lasted 4–7 days, depending on the rat. Following this initial acquisition phase at FR1, the ratio to obtain the drug was increased geometrically every other day: FR3 (3 nose pokes are required to obtain 1 infusion of morphine), FR6, FR12, FR24, FR48, and FR96. This allows for analysis of drug consumption as a function of “price” using demand curves for behavioral economics, which can estimate the motivation to obtain drugs and their “addictive potential” (20). For this, data from each rat were fitted to the equation log Q = log Q_0_ + k (e ^–α(Q0^ ^C)^ – 1), where Q represents consumption (number of self-infusions), and Q_0_ represents the level of consumption at the lowest price. C represents price for the drug (i.e., the ratio), and k is set to a constant accounting for the estimated range of consumption in logarithmic units (k = 4 in these studies). The symbol α is the relative change in consumption across changes in price (ratio). Behavior is considered “inelastic” when consumption is insensitive to price (i.e., the curve is flat, and consumption is maintained despite increases in price), and it switches to “elastic” when consumption is sensitive to price (i.e., when the curve is steep and consumption declines with increases in price). A drug is deemed to have a higher addictive potential when it has low elasticity and when the switch from inelastic to elastic occurs at high prices (37, 38). Using this curve, we can also calculate *P*_max_, which is calculated as 0.65/(Q_0_ × α × k^1.191^); greater *P*_max_ indicate greater effort to obtain the reward (38). We measured the number of responses in the active and inactive holes and the number of self-infusions. We also compared groups for their average intake in the consumption-price curves by measuring α (elasticity) and *P*_max_.

#### Progressive ratio testing (within-session).

Rats were tested for self-administration in a within-session progressive ratio test in which the ratio to obtain one infusion increased semi-logarithmically as 1, 2, 4, 6, 9, 12, 15, 20, 25, etc., within a single 4-hour self-administration session (21). This allows testing for the “breaking point” (the highest ratio reached to obtain one infusion).

### Induction of morphine dependence

Morphine dependence in mice was induced by repeated i.p. injections (10 mL/kg) of morphine administered at increasing doses, over 5 days (day 1: 20 mg/kg, day 2: 40 mg/kg, day 3: 60 mg/kg, day 4: 80 mg/kg, day 5: 100 mg/kg). Injections of each dose were administered twice daily, 6 hours apart. This dosing was chosen because it produces spontaneous opioid withdrawal and withdrawal-induced mechanical hypersensitivity in mice (19).

### Conditioned place aversion and somatic signs of morphine withdrawal

The place conditioning apparatus was an acrylic box enclosed in light- and sound-attenuating, ventilated chambers (MED Associates). The box had 2 compartments separated by a removable sliding door. Compartment 1 had a floor composed of metal bars (0.24 cm) placed in parallel series. Compartment 2 had a floor composed of a metal grid mesh (0.63 cm). The dimensions of each compartment were 13.5 × 27 × 17 cm. Infrared light beams and photodetectors located along the walls of the box measured general activity and location of the mouse in each compartment, which were recorded using Activity Monitor version 7.0.5.10 software (Med Associates). On day 0 (pretest), drug-naive mice were acclimated for 15 minutes to the place conditioning apparatus without the sliding door, so they had free access to both compartments of the apparatus. This established a baseline side preference, based on the amount of time spent in each compartment. Mice that exhibited > 75% preference for a certain compartment were removed from the study. Then, mice were distributed in each experimental group according to their preference scores, to ensure similar values across groups. Then they received repeated injections of saline (10 mL/kg, i.p.) or morphine (20–100 mg/kg, i.p.), twice a day, for 5 days. On day 6 (conditioning day), mice were pretreated with vehicle (10 mL/kg, i.p.) or CP612 (5, 10, 20, or 40 mg/kg, i.p.), followed by a single injection of morphine (100 mg/kg, i.p.) 4 hours later. Two hours after this last injection of morphine, mice were administered naloxone (5 mg/kg, i.p.), to precipitate withdrawal, and were immediately confined to 1 side of the conditioning apparatus for 20 minutes. During this conditioning period, mice were filmed (Raspberry Pi Camera) to assess somatic signs of withdrawal (39). Given the limited number of cameras and the poor quality of some videos, we were only able to film a subset of mice. The following behaviors were given a score of 1 per occurrence and episode: body shakes (paw tremors and wet dog shakes), teeth chatter, rearing, hopping, wall climbing, and backpedaling. These scores were summed to obtain a global withdrawal score and were obtained from an independent observer who was blind to the experimental conditions. A subset of videos was scored by 2 observers, to ensure internal validity (Cronbach’s α = 0.980, Correlation _Observer_ *r* = 0.97, slope 1.15, *P* < 0.005). On day 7 (test day), mice were placed in the conditioning apparatus with free access to both compartments, for 15 minutes. We recorded the time spent in each compartment.

### Statistics

Kinase assays used 11-point concentration-response curves and results were analyzed by nonlinear regression using Prism 9 (GraphPad Software) to determine IC_50_ values. Other analyses were performed on Prism 9 and Statistica (Tibco Software). Scores for pain threshold, conditioned place preference, and self-administration were analyzed by ANOVA using the following factors. Between-subject factors included PKC inhibition (vehicle, CP612 at different doses), chemotherapeutic treatment (vehicle, paclitaxel), and opioid treatment (morphine, saline); within-subject factors (i.e., repeated measures) included time (different times or days of testing) and hole (active hole, inactive hole). We used a Tukey’s test for post hoc comparisons across groups and Dunnett’s test for post hoc comparisons with own pain thresholds at baseline values in the pain studies. Somatic withdrawal scores were analyzed with a Kruskal-Wallis test due to the nonparametric nature of those data, followed by nonparametric post hoc comparisons according to Siegel and Castellan (40). A *P* value less than 0.05 was considered significant.

### Study approval

All procedures were done following the *Guide for the Care and Use of Laboratory Animals* (National Academies Press, 2011) and were approved by the IACUC of The University of Texas at Austin (AUP-2019-00189 and AUP-2022-00116) and of UCSF (AN204716-00B).

### Data availability

Underlying data can be found in the Texas Data Repository (https://doi.org/10.18738/T8/UZVBBW) and in the Supporting Data Values file, which include values underlying graphed data and reported means presented in the main text and supplemental material.

## Author contributions

ROM, JDL, SFM, and MM conceived the study and designed the experiments. HCDK and SFM designed the synthesis of compound CP612. CF performed the kinase assays. AGF, IJMB, SD, NMR, and MM conducted animal experiments. NAC and PML conducted the in vitro ADME studies. AGF, IJMB, SD, HCDK, NAC, PML, CF, and MM analyzed data in collaboration with ROM, JDL, and SFM. AGF, ROM, JDL, SFM, and MM wrote the manuscript.

## Supplementary Material

Supplemental data

Supporting data values

## Version 1 04/22/2025

Electronic publication
