# Supplementary material for: A small molecule PKCε inhibitor reduces hyperalgesia induced by paclitaxel or opioid withdrawal
Source: JCI Insight. 2025 Apr 22;10(8):e186805. doi: 10.1172/jci.insight.186805 (PMC12016938; doi:10.1172/jci.insight.186805)
Supplement: Supplemental data [file jciinsight-10-186805-s053.pdf]

Table S1. Physicochemical properties of CP612

Compound	Plasma stability t½ (min)	Plasma stability Cl (µL/min)	Microsomal clearance t½ (min)	Microsomal clearance Cl (µL/min)	Plasma protein binding (% bound)
CP612	90.17 ± 6.75	0.46 ± 0.04	628.74 ± 78.56	2.23 ± 0.29	89.78 ± 1.65
Procaine	1.65 ± 0.09	25.29 ± 1.35			
Procainamide	1174.11 ± 337.30	0.038 ± 0.013			
Testosterone			12.73 ± 1.37	109.81 ± 11.69	93.87 ± 0.20
Metoprolol					10.16 ± 1.46

t½: half-life; Cl: clearance.

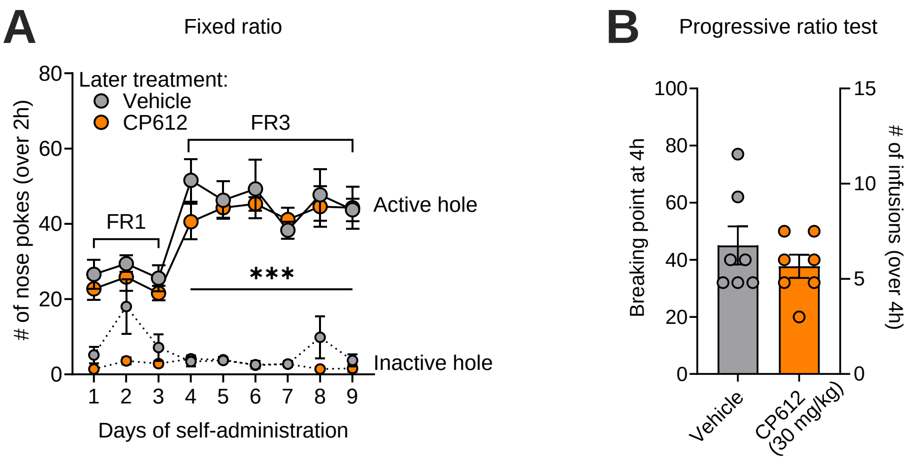

Figure S1. Self-administration of morphine, after administering CP612 18h prior. (A) Rats acquired self-administration of morphine (500 µg/kg/i.v. infusion), attested by greater responding in the active hole vs. the inactive hole [ANOVA $F_{\text{Hole type}}$ (1,12) = 264.68, p < 0.001] and this occurred to a similar extent in rats that would later receive vehicle or CP612 [ANOVA $F_{\text{Hole type x PKC inhibition}}$ (1,12) = 0.012, p > 0.91]. The average intake of morphine was 13.4 infusions, and this intake was similar in rats that would later receive vehicle or CP612 [ANOVA $F_{\text{PKC inhibition}}$ (1,12) = 0.182, p > 0.79; $F_{\text{Time x PKC inhibition}}$ (8, 96) = 0.47, p > 0.88]. **(B)** During the progressive ratio tests, the ratio to obtain an infusion was increased progressively within a self-administration session. Administration of CP612 (30 mg/kg, i.p.) 18h prior, did not modify self-administration. This was measured as breaking point (highest ratio reached to earn an infusion of drug) and number of self-infusions [ANOVA $F_{\text{PKC inhibition}}$ (1,12) = 0.87, p > 0.36 and = 0.80, p > 0.39 for breaking point and self-infusions, respectively]. Data are mean and SEM from n=7 rats/group; dots are scores from individual rats. ***p < 0.001 compared with the inactive hole using Tukey's post-hoc test.

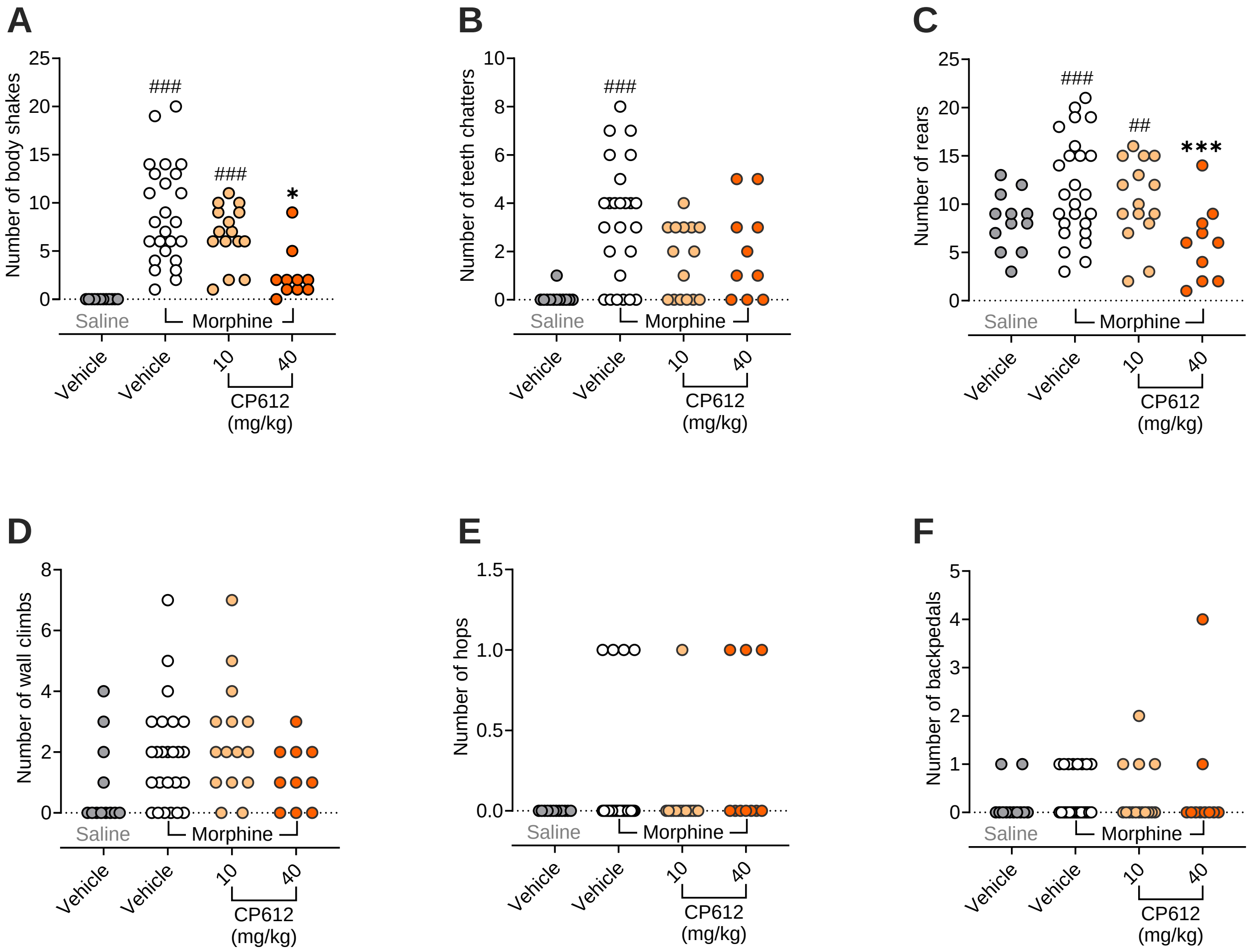

Figure S2. Behavioral scores of morphine withdrawal precipitated by naloxone. Male mice received repeated injections of saline (10 mL/kg, i.p.) or morphine (20-100 mg/kg, i.p.) twice daily for 5 days. On day 6, during the conditioning day, they were pretreated with vehicle control (10 mL/kg, i.p.) or CP612 (10 mg/kg or 40 mg/kg, i.p.) followed by an injection of morphine (100 mg/kg, i.p.) 4h later. After 2h, they received an injection of naloxone (5 mg/kg, i.p.) to precipitate withdrawal. Withdrawal scores were analyzed with Kruskal Wallis test, due to the non-parametric nature of the data. Compared with mice that received repeated injections of saline, mice that received repeated injections of morphine showed withdrawal signs precipitated by naloxone for **(A)** body shakes [H (3, n = 62) = 37.400, p < 0.001], **(B)** teeth chatter [H (3, n = 62) = 18.238, p < 0.001], **(C)** rearing [H (3, n = 62) = 11.49, p < 0.01], and **(D)** wall climbing [H (3, n = 62) = 7.849, p < 0.05], but not **(E)** hopping [H (3, n = 62) = 4.802, p = 0.187], or **(F)** backpedaling [H (3, n = 62) = 0.920, p = 0.821]. For treatments with significant effects, only the high dose of CP612 (40 mg/kg, i.p.) attenuated some of these withdrawal signs (##p < 0.01 and ###p < 0.001 compared with saline + vehicle; *p < 0.05 and ***p < 0.001 compared with morphine + vehicle). Dots are scores from individual mice.
